# Effects of the *FecL* major gene in the Lacaune meat sheep population

**DOI:** 10.1186/1297-9686-46-48

**Published:** 2014-08-12

**Authors:** Pauline Martin, Jérome Raoul, Loys Bodin

**Affiliations:** 1 Institut de l’Elevage, BP 42118, 31321 Castanet Tolosan cedex, France; 2 INRA, UMR1388 Génétique, Physiologie et Systèmes d’Elevage, F-31326 Castanet-Tolosan, France; 3 Université de Toulouse INPT ENSAT, UMR1388 Génétique, Physiologie et Systèmes d’Elevage, F-31326 Castanet-Tolosan, France; 4 Université de Toulouse INPT ENVT, UMR1388 Génétique, Physiologie et Systèmes d’Elevage, F-31076 Toulouse, France

## Abstract

**Background:**

The major prolificacy gene *FecL* was first described in the Lacaune sheep meat breed Ovi-Test in 1998. A few studies estimated the effect of this gene on prolificacy but little data is available. In 2010, the Ovi-Test cooperative started genotyping *FecL* in all of their replacement ewe lambs. Thanks to the large amount of genotyping data that is available now, gene effects on litter size and other relevant traits can be estimated more accurately.

**Methods:**

Our study included 5775 ewes genotyped since 2010 and 1025 sires genotyped since 2002. Performances and pedigrees were extracted from the French national database for genetic evaluation and research. Analysis of the effect of the gene on different traits was based on linear or threshold genetic animal models using the ASReml software.

**Results:**

The female population was composed of 71% homozygous wild type ewes (++), 27% heterozygous ewes for the *FecL* mutation (L+) and 2% homozygous mutant (LL) ewes. On average, L + ewes produced 0.5 more lambs per lambing than ++ ewes. The *FecL* gene not only affected the mean litter size but also its variability, which was lower for ++ than for L + ewes. Fertility after insemination was higher for L + ewes than for ++ ewes. Lambs from ++ dams were heavier (+300 g) than the lambs of L + dams and the mortality of twin lambs born from ++ dams was lower than those from L + dams. In addition, bias in estimated breeding values for prolificacy when ignoring the existence of this major gene was quantified.

**Conclusions:**

The effect of the *FecL* gene on prolificacy was estimated more accurately and we show that this gene affects both the mean and the variability of litter size and other traits. This paper also shows that ignoring the existence of this major gene in genetic evaluation of prolificacy can lead to a large overestimation of polygenic breeding values.

## Background

The Lacaune sheep breed is the main breed raised in France, with approximately 1.2 million ewes. There are different strains of this breed, depending on the production purpose (dairy or meat). In 1975, the artificial insemination (AI) cooperative Ovi-Test designed a selection program to increase prolificacy of sheep bred for meat production [1]. Prolificacy is considered as a difficult trait to select for because of its low polygenic inheritance. However, the improvement in prolificacy was higher than expected with, between 1975 and 1996, an increase from 1.28 to 1.98 for ewe lambs mated at approximately 11 months of age in June and July [2]. This rapid response to selection, together with several other observations, suggested a non-polygenic inheritance and the segregation of a major gene in this population [2].

Since 1982 and the first evidence of a major gene for prolificacy in Booroola Merinos [3,4], various studies demonstrated or suspected the existence of major genes in other breeds [5,6]. In the Lacaune meat sheep population, two major genes that affect prolificacy have been identified: (1) the *FecX*^*L*^ mutation (C53Y) of the *BMP15* gene, which is located on the X chromosome and results in a high ovulation rate (1.5 additional ova per ewe) in heterozygous ewes and sterility in homozygous mutated ewes [7] and (2) the *FecL* gene that is located on chromosome 11 and for which two SNPs (single nucleotide polymorphisms) close or within the *B4GALNT2* gene are candidate causal mutations [8,9]. The *FecL* gene has two alleles and each copy of the mutant allele (designated L) increases ovulation rate by approximately 1.5 additional ova compared to the wild allele (designated +). In 2006, allele frequencies of both genes were estimated in the Lacaune sheep population by genotyping the males at the AI centre [8] and the proportion of ewes with the mutant allele was estimated at 15% for *FecX*^*L*^ and 45% for *FecL*.

Homozygous mutant animals for either of these genes are not adapted to breeders’ needs because of the sterility of *FecX*^*L*^ homozygous ewes and excessive prolificacy of *FecL* homozygous ewes. Moreover, some on-farm observations and experimental data [10] showed that the prolificacy of double heterozygous animals reached levels similar to that of homozygous *FecL* animals. Because of the relative frequencies of each of these genes, the sterility induced by the *FecX*^*L*^ mutation in the *BMP15* gene, the excessive prolificacy of homozygous *FecL* ewes, and the poor prolificacy of wild type ewes, the Ovi-Test cooperative chose to remove animals that carried the *FecX*^*L*^ mutation from its population and to manage the *FecL* gene at the heterozygous state. Thus, since 2002 all sires are genotyped for both genes and, since 2010 every replacement ewe lamb is genotyped for the *FecL* gene.

In 2006, the first estimate of the effect of the *FecL* mutation on prolificacy was reported [10], but only a few ewes from commercial flocks were genotyped at the time and the study compared the daughters of carrier and non-carrier sires. The recent systematic genotyping program has provided a large amount of data. Using this data, the present study aimed at estimating the effect of the *FecL* mutation on prolificacy in a large and unselected sample. Potential effects on fertility after AI, lamb growth and mortality were also analysed to check if the mutation affects these traits directly, since they are important traits for the breeders. In addition, we quantified the bias in estimates of the additive polygenic values since, currently the evaluation process of estimated breeding values (EBV) considers that genetic effects are transmitted according to Fisher's infinitesimal model [11].

## Methods

### Ovi-Test breeding program

The cooperative nucleus consisted of 26 breeder flocks, with 430 ewes per flock on average (from around 150 to 1000), and a total of 11 201 ewes in June 2013. Most flocks perform three lambings every two years. This breeding program is on-going.

Male candidates used in the nucleus are preselected when they are about 45 days old and are genotyped for *FecL* when they enter the central testing station for own performance test. Then, they are selected on their own performances for growth, fatness and conformation. Those with less favourable performances (about 50%) are used for natural mating in the nucleus, sold or slaughtered, while the best males continue on in the selection process and undergo a progeny test set up on-farm by AI. The females used as replacement ewes are chosen among the AI-born lambs (about 40% of the lambs result from AI in the nucleus). The first mating for these replacement ewes is a natural mating (ewe lambs are not artificially inseminated in this breeding program).

In order to manage the *FecL* gene within the population to address the breeder’s goal of optimal prolificacy, the Ovi-Test cooperative imposed various mating rules. The objective is to obtain a nucleus consisting of 50% heterozygous ewes (designated L+) and 50% homozygous wild-type ewes (designated ++), and to minimize the production of homozygous mutant ewes (designated LL). When AI is used, L + and LL ewes must be mated to ++ rams, and ++ females to L + rams. Only ++ rams can be used for natural mating.

### Genotyping

From 2004 to 2009, the presence of the *FecL* mutation was predicted by a combination of alleles at three microsatellite loci that determine an L-haplotype that was never detected on wild-type chromosomes [2,8]. Since 2009, *FecL* genotyping is based on SNP DLX3:c.*803A > G (GenBank accession number FJ654646), which is in high linkage disequilibrium with the *FecL* mutation (<1% recombination) and located in the 3′ UTR sequence of the *DLX3* gene [8]. Until recently, it was the most efficient marker that INRA could transfer to Aveyron-labo, the commercial genotyping company that performs the genomic analyses for the Lacaune breed.

### Origin and selection of data

All data (performances for AI result, litter size, lamb mortality and growth; pedigree over five generations) were extracted from the national database for genetic evaluation and research managed by the Institut de l’Elevage (French Livestock Production Institute) and the CTIG (Centre de Traitement de l'Information Génétique, Jouy-en-Josas, France). Only females born after 2010 (when consistent genotyping of replacement ewes began) and sires genotyped since 2002 and used in the nucleus were retained (n = 5775 and 1025, respectively). However, too few LL ewes were available to calculate accurate estimates for this genotype and they were removed; thus only ++ and L + genotypes were compared.

### Statistical analysis

#### ***Genotype frequencies***

Allele and genotype frequencies were estimated separately for female and male populations. Potential changes in frequencies between years, as well as Hardy-Weinberg equilibrium, were tested with a Chi-squared test.

#### ***Analysis of the gene effect on different traits***

In addition to the genotype of the ewe, many factors can affect the four traits analysed and they were defined as similar as possible to those of the national genetic evaluation system. However, not all factors were tested for each model due to the structure of the data. The list of factors tested for each trait and included in the final analysis is in Table 1. The fixed effects and all one-way interactions with biological meaning included in the models were initially selected step-by-step using nested models that were compared with the likelihood ratio test. These models were fitted using the mixed procedure of SAS^®^ V9.2, while final analyses were performed using the ASReml software [12].

**Table 1:** Fixed effects tested in the analysis of the different traits

**Ewe effects**	**Litter size**	**AI fertility**	**Liveweight at 30 days**	**Mortality**
Genotype	*	*	*	*(only for twin litters)
Flock	*	*	*	*
Year of lambing	x	-	*	x
Season of lambing	x	-	*	-
Month of lambing	*	-	*	*
Season of AI	-	x	-	-
Birth type	x	x	*	x
Rearing mode	*	*	*	*
Rearing type	*	x	*	x
Interval since last lambing	*	*	*	-
LS at previous lambing	*	-	*	-
LS suckled at previous lambing	-	*	-	-
Age at 1^st^ lambing (3 classes)	*	-	*	x
Lambing rank	*	-	*	*
Oestrus type (natural, hormonal treatment)	*	-	-	-
**Lamb effects**				
Sex			*	-
Birth type			*	*
Rearing type			*	-

LS = litter size, * = significant (P < 0.05); x = not significant (P > 0.05); - = not tested; for litter size and liveweight at 30 days, the effects of rearing mode and rearing type of the ewe, lambing rank, age at first lambing, litter size at previous lambing, and interval since last lambing were combined in a single effect called “physiological status of the dam”; for liveweight at 30 days, the effects of flock, year of lambing, and season of lambing were combined in a single effect renamed flock.

##### 

**Litter size** The distribution of raw litter size (LS) per genotype is in Table 2. A total of 4260 records from ++ and L + ewes were used. Litter size is a categorical trait that has to be analysed by a threshold model [13], assuming the existence of an underlying normal variable and a set of thresholds that determine the observed categories. In this study, to avoid extreme case problems [14], litters of more than three lambs were clustered into a single class. Once the final model was chosen, fixed effects and variances were estimated with a threshold animal mixed model [15]. The model was fitted with the fixed effects chosen as described previously (Table 1). The model considered a standard normal underlying variable N(0, 1) and a set of thresholds shared both by ++ and L + genotypes, as well as their interaction with genotype, resulting in a set of thresholds for each genotype ({${\hat{\tau }}^{++}$} and {${\hat{\tau }}^{L+}$}). Since a drastic relationship between mean and variance exists for sheep prolificacy [16], the comparison of variances on the observed scale was not direct but based on the predicted distribution of LS for each genotype through a back transformation to the observable scale using the estimated thresholds for each genotype ($\left\{{\hat{\tau }}^{i}\right\},\;i=++,L+$). The probability of response in the *j*^*th*^ category for genotype *i* was computed for an underlying variable N(*η*, 1) as: ${\hat{\Pi }}_{j}^{i}=\Phi \left({\hat{\tau }}_{j}^{i}+\eta \right)-\Phi \left({\hat{\tau }}_{j-1}^{i}+\eta \right)$_,_ setting $\tau_{0}^{i}=-\infty$ and $\tau_{4}^{i}=+\infty$.

**Table 2:** Distribution (number and proportion) of observed litter size by genotype in the whole dataset

**Genotype**	**1**	**2**	**3**	**4**	**5**	**6**	**Total**
++	1528	2176	322	39	5	1	4071
	0.37	0.53	0.08	0.01	0.001	ϵ	
L+	291	679	374	83	10		1437
	0.20	0.47	0.26	0.06	0.01		
LL	17	32	24	10	2	2	87
	0.20	0.37	0.28	0.11	0.02	0.02	

Then, mean and variance were estimated for a given *η* as:

$${\hat{\mu }}_{\text{obs}}^{i}\left(\eta \right)=\sum_{j=1}^{4}j{\hat{\Pi }}_{j}^{i}\left(\eta \right)$$

and ${\hat{\sigma }}_{\text{obs}}^{{}^{2}i}\left(\eta \right)=\sum_{j=1}^{4}j^{2}{\hat{\Pi }}_{j}^{i}\left(\eta \right)-{\left({\hat{\mu }}_{\text{obs}}^{i}\left(\eta \right)\right)}^{2}$.

These calculations were done for several values of *η*. Another more illustrative method is proposed in the Appendix. It consists in a transformation of the threshold sets estimated for ++ and L + ewes into a common set for both genotypes and instead of fixed parameters (*η* = 0, *σ*^2^ = 1) for the underlying variable to give specific values for each genotype. In this case, the variance of the underlying variable is higher for L + than for ++ ewes. Both methods provide similar evidence that the *FecL* gene affects both the mean and the variability of litter size. Polygenic heritability was estimated by the ratio of the animal variance component to the total variance on the underlying scale, which was equal to the sum of the animal variance component and the residual variance; that last variance was set to 1 in this threshold model.

##### 

**Artificial insemination fertility** Since the mutation affects the number of ovulations, it was relevant to investigate whether it also affects their quality, which can be assessed indirectly by female fertility. Since conditions of natural mating are not well documented in our database, in this study we focused on the effect of the gene on AI fertility. Because ewe lambs were not artificially inseminated in the nucleus, the data used to evaluate the effect of genotype on the success of AI included only a particular sub-sample of ewes. These ewes were more than 14 months old and were primiparous or multiparous when inseminated. Because the number of inseminated ewes that had suckled three or more lambs during their last litter was very small, they were discarded from the analysis. The fixed effects considered for this trait are shown in Table 1. For this sub-sample, the interval of time since the last lambing was considered as a covariate and the season was divided into three periods (November to March, April, and May to July). Finally, for the 1206 available ewes, the AI was considered as fertile if lambing occurred between 142 and 152 days after it was performed. AI success is a discrete variable for which genotype and other fixed effects were estimated using a threshold animal mixed model.

##### 

**Lamb weight at 30 days of age** The aim of this analysis was to observe the effect of dam genotype on the weight of lambs at 30 days of age (30dLW), which is used as a maternal capacity selection criterion in the Ovi-Test breeding program. It was carried out only in 17 flocks in which all lambs were weighed at about 30 days of age. Because some lambs were not weighed at exactly 30 days, the 30dLW was calculated by linear regression using the actual weight at the given age and a fixed birth weight for each lamb category (sex, litter size). The variable 30dLW was analysed for 3829 lambs using a linear animal mixed model, with fixed effects as described in Table 1.

##### 

**Mortality from birth to 30 days of age** The effect of dam genotype on lamb survival was studied by measuring lamb mortality from birth to 30 days of age. Sex could not be included as a fixed effect because the gender of stillborn lambs was unknown. The rearing type was discarded for similar reasons. This binary trait was analysed for 8338 lambs using a threshold animal mixed model.

### Estimated breeding values for prolificacy

Bias in estimates of additive polygenic values for prolificacy of the 2711 genotyped females with lambing records was estimated by comparing BLUP (best linear unbiased prediction) of their breeding values from a threshold animal mixed model which did (EBV|_FecL_) or did not (EBV) include the genotype effect as a fixed effect. Comparison of these EBV between ++ and L + ewes was done by a simple ANOVA. Bias due to ignoring the genotype effect in the evaluation of breeding values were assessed within genotypes by comparing the difference between EBVand EBV|_FecL_ with a paired Student's t-test and the slopes of the regressions of EBVon EBV|_FecL._

## Results

### Genotype frequencies

Genotype frequencies of the female genotyped population are in Table 3 by year of birth and genotype frequencies of the sires are in Table 4. The frequency of the L allele in the replacement females was 15.3%. For these females, the genotype frequencies were very stable and did not depend on year of birth (P-value = 0.50); globally they were not in Hardy-Weinberg equilibrium (P = 0.05). Moreover, genotype frequencies differed between the farms, with values ranging from 62.1 to 81.7% (average [standard deviation] = 70.5 [5.2]) for ++ ewes, from 17.9 to 34.7% (27.3 [4.6]) for L + ewes, and from 0.0 to 6.3% (2.15 [1.4]) for LL ewes. The frequency of the mutation was slightly higher in sires genotyped between 2002 and 2013 (17.4%) than observed for ewes, which resulted in slightly different genotype frequencies compared with the females (P = 0.02) but genotype frequencies satisfied Hardy-Weinberg equilibrium (P = 0.99).

**Table 3:** Genotype frequencies for the 5775 genotyped females between 2011 and 2013

**Genotype**	**Number**	**Overall frequency**	**Frequencies for ewes born in**		
			**2011 (n = 2064)**	**2012 (n = 2033)**	**2013 (n = 1678)**
++	4123	71.4	71.1	72.7	70.1
L+	1536	26.6	26.8	25.3	27.9
LL	116	2.0	2.1	2.0	2.0

**Table 4:** Genotype frequencies for the 1025 genotyped sires from 2002 to 2013

**Genotype**	**Number of sires**	**Overall frequency**
++	699	68.2
L+	295	28.8
LL	31	3.0

### Litter size (LS)

**Figure 1:**
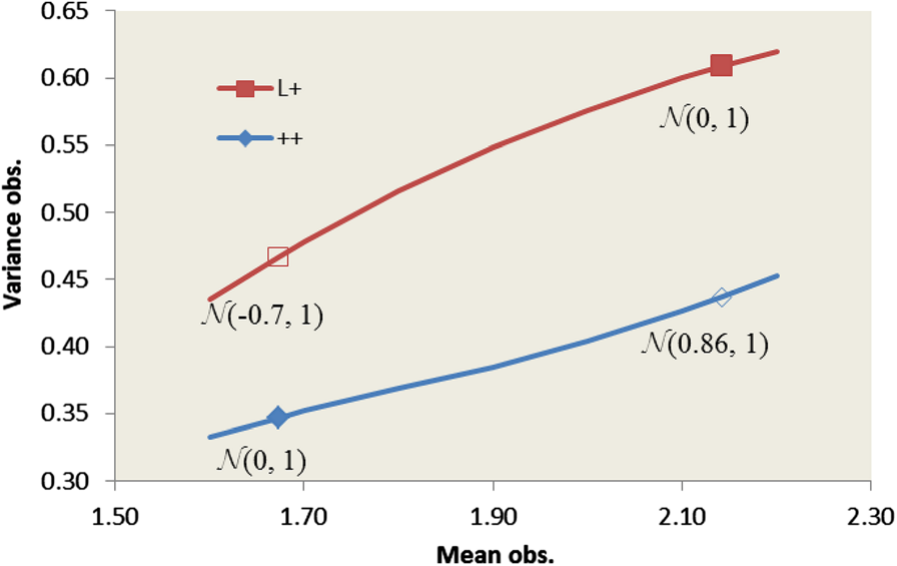
**Relationship between predicted mean of litter size on the observable scale (Mean obs.) and predicted variance (Variance obs.) for each genotype according to the mean of the underlying variable**^**†**^**. **^†^ Predicted mean and variance of litter size on the observable scale were calculated for each genotype by combining the specific set of thresholds {τ^++^} and {τ^L+^} with an underlying variable Ν(η, 1) for which the mean η varied from -1 to +1. Plain markers correspond to the underlying variable Ν(0, 1) of the output of the threshold model.

The linear model run to select the significant effects showed highly significant effects for genotype (P < 0.001), oestrus type (P < 0.01), flock (P < 0.001), month of lambing (P < 0.001) and the composite effect or physiological status of the dam at lambing (P < 0.001). In the threshold animal mixed model, the interaction between thresholds and genotypes was significant (P < 0.01) and the likelihood ratio test showed a better data fit when a separate set of thresholds was considered for each genotype ({τ^++^} and {τ^L+^}), compared considering a common set of thresholds {τ^c^} and an effect of genotype on the mean of the underlying variable. As expected, estimates of the thresholds were lower for the prolific L + ewes than for the ++ ewes (Table 5), but the sets of thresholds could not be simply translated between the genotypes. Since the model was not linear, predictions were obtained for a given combination of levels of fixed effects. Predictions of LS corresponded to the actual estimated value when the mean *η* of the underlying variable was set to 0 for each genotype. The difference in means on the observable scale was then equal to 0.47 lambs per lambing. Other values of *η* led to theoretical distributions of LS. Figure 1 shows that regardless of the value of *η* (within a range which gives a mean on the observable scale between 1.60 and 2.20), the variance on the observable scale was always higher for the L + than for the ++ genotype. In particular, an underlying variable N(0.86, 1) associated with the thresholds {${\hat{\tau }}^{++}$} led to the same mean on the observed scale than an underlying variable N(0, 1) associated the thresholds {${\hat{\tau }}^{L+}$} but resulted in a much higher variability for the L + genotype (+39%). Another way to show that the *FecL* gene has an effect on both the mean and variance of the trait is presented in the Appendix.

**Table 5:** **Estimated thresholds for each genotype and resulting distribution, mean and variance of litter size (LS) according to the parameters η and σ**^**2 **^**of the underlying variable**

**Estimated thresholds**	**++**	++^*^	**L+**	L+^*^
τ_1_	-0.287		-0.849	
τ_2_	1.581		0.539	
τ_3_	2.711		1.691	
Underlying variable parameters				
η	0	0.86	0	-0.70
σ^2^	1	1	1	1
Resulting parameters on the observable scale				
μ_obs_	1.67	2.14	2.14	1.67
σ^2^_obs_	0.35	0.44	0.61	0.47
%LS1	38.71	12.56	19.79	44.23
%LS2	55.60	63.88	50.71	45.07
%LS3	5.36	20.34	24.95	9.87
%LS4+	0.34	3.21	4.54	0.83

Values in columns ++ and L + correspond to the output of the threshold model: thresholds (τ1- τ3), mean and variance of the underlying variable (η, σ^2^) and the resulting mean and variance (μ_obs,_ σ^2^_obs_) of the litter size percent (%LS1 - %LS4+) on the observable scale. Values for ++* and L + * columns correspond to the ++ and L + thresholds and a shift of the mean η of the underlying variable which would provide mean of L + and ++ LS on the observable scale, respectively.

The polygenic genetic variance was estimated at 0.156, which resulted in a polygenic heritability in the underlying scale of 0.14 (s.e. = 0.020). As expected, estimates of additive genetic variance and heritability were higher when the model did not include the effect of genotype (genetic variance = 0.221; heritability = 0.18 (s.e. = 0.02)).

### AI fertility

Genotype of the ewe (P = 0.016), flock (P < 0.0001), rearing mode (P = 0.05), interval since the last lambing (P = 0.04), and number of lambs suckled in the previous litter (P < 0.0001) had a significant effect on the AI fertility. The average fertility observed following AI was high (0.74) and higher for L + ewes (0.79) than for ++ ewes (0.72). Based on results of the model, L + ewes were significantly more fertile than ++ ewes (P = 0.016) and fertility decreased as the number of lambs suckled in the previous litter increased (P < 0.0001). The difference between genotypes was 0.3 on the underlying scale. Predictions on the observed scale were obtained for a given combination of levels of fixed effects. For ewes in a flock with an average effect, rearing a single lamb, and having suckled two lambs at the previous lambing, the difference between genotypes was 11% points on the observed scale: 62% for ++ ewes and 73% for L + ewes.

### Lamb weight at 30 days of age

Genotype of the dam, flock, month of lambing, physiological status and birth type of the dam, and sex, birth and rearing type of the lamb had significant effects on 30dLW (P < 0.001). Male lambs were heavier than female lambs and the weight decreased when the birth type or the rearing type increased. When all possible sources of variation linked to dam prolificacy but independent of the *FecL* genotype were taken into account, lambs of ++ dams were about 300 g heavier at 30 days of age (P < 0.001) than lambs of L + dams. This difference accounted for about 3% of the lamb’s weight at this age.

### Lamb mortality

Flock (P < 0.0001), month of lambing (P = 0.04), rearing type of the dam (P = 0.05), and lambing rank (P < 0.0001) had significant effects on the risk of mortality. The effect of genotype of the dam was not significant (P = 0.13) but the interaction between birth type of the lamb and dam genotype was significant (P < 0.0001). However, the difference between genotypes was significant only for twin litters (P < 0.001). In order to better characterize this effect, the model was run again for twin lambs only. The probability of death was higher for twin lambs of L + dams than for those of ++ dams and the estimated difference between the genotypes was 0.19 on the underlying scale. For a particular combination of fixed effects (average flock, lambing in May, first lambing), these estimates converted to probabilities of 0.155 and 0.114, for L + and ++ dams respectively.

### Estimated breeding values for prolificacy

When the genotype effect was included in the model, there was no significant difference between estimated additive polygenic breeding values (EBV|_FecL_) of ++ and L + ewes (Table 6), whereas when this effect was ignored, estimated polygenic breeding values (EBV) were significantly lower for ++ than for L + ewes (P < 0.001). The latter difference was greater than 1 standard deviation unit of the EBV|_FecL_ (σ = 0.192). Ignoring the genotype effect led to a very small underestimation of additive polygenic values for ++ ewes and a large overestimation for L + ewes of about 1 standard deviation unit of the EBV|_FecL_ (Table 6). Furthermore, the slope of the regression of EBV on EBV|_FecL_ showed that the small underestimation of additive polygenic values was almost constant for the ++ genotype (β = 1.07, standard error = 0.01) while the overestimation observed for the L + genotype increased slightly when the additive polygenic value increased (β = 1.30, standard error = 0.02).

**Table 6:** Means and standard deviations of estimated breeding values (EBV) for prolificacy of the 2710 females with LS by genotype

**Breeding values**	**++ (n = 2009)**			**L + (n = 701)**			**(++) – (L+)**
	**μ**	**σ**	**Paired T test**	**μ**	**σ**	**Paired T test**	**F test**
EBV	0.102	0.227		0.295	0.253		P < 0.001
EBV|_FecL_	0.125	0.196		0.110	0.179		P = 0.07
(EBV) – (EBV|_FecL_)	-0.023	0.084	P < 0.001	0.185	0.115	P < 0.001	P < 0.001

Estimates were based on a threshold animal mixed model that included the effect of the gene (EBV|_FecL_) or not (EBV); tests between genotypes were done by ANOVA, and between breeding values with a paired T test.

## Discussion

### Allele and genotype frequencies

The genotype frequencies observed in females were slightly different from those estimated in 2006 (Ovi-Test, personal communication). Two reasons could explain these differences. First, the study performed in 2006 was based on a small number of genotyped animals. Moreover, before consistently genotyping replacement ewes, the cooperative excluded animals that were genetically estimated as very prolific. Males with an EBV for prolificacy greater than 1.5 standard deviation units above the mean, and ewes with an EBV greater than 3 standard deviation units above the mean were automatically discarded from the breeding program. This strategy, which was used from 1996 to 2010 to avoid animals that were too prolific, probably reduced the proportion of L + animals.

The high stability of genotype frequencies observed since 2011 is not really surprising, despite the cooperative’s aim to achieve 50% of L + ewes. A substantial number of genotyped ewes ready for insemination were available only in 2012 and a too sudden change in genotype frequencies would not be suitable for the breeders.

The considerable variation in genotype frequencies observed between flocks was somewhat surprising since by obligation, all replacement ewes in the nucleus originated from collective AI sires. This rule was set up at the beginning of the selection scheme in 1975 and is still compulsory. Therefore, these differences in genotype frequencies could result from the different strategies implemented by individual breeders to select their replacement ewes. Some breeders may select ewe lambs born as triplets, and most probably born from carrier dams, whereas others may avoid keeping these smaller ewe lambs. These counter-selection procedures, together with the higher mortality of progeny born from prolific carrier ewes, also explain the deviation from Hardy-Weinberg equilibrium observed for females. In contrast, the male selection strategy is imposed by the cooperative as a group strategy. It slightly favours the carriers, counterbalancing their higher mortality, which brings the male genotype frequencies back to Hardy-Weinberg equilibrium.

### Effects on prolificacy

The effect of the *FecL* mutation on prolificacy found in the present study agrees with the first observations previously reported [10]. As for other major genes that affect prolificacy in sheep, the effect is lower on this trait than on ovulation rate [2,3,6,17-20]. The increase of about 0.5 lambs per lambing for one copy of the mutant allele is of the same order of magnitude as the effect of all other known major genes for prolificacy. However, it is slightly lower than the prolificacy increase previously reported for Booroola ewes (+0.9 to 1.2 lambs per litter) that carry a single copy of the mutant Booroola allele [21]. The estimate of the effect of *FecL* on prolificacy is also similar to the lowest effect of the Booroola gene stated in the extensive review performed by Fogarty [22]. It is also lower than the effect of a single copy of the *BMP15* gene that contains either the Hanna [23] or Inverdale [24] mutations, the Belclare or Galway mutations [19], or the Rasa Aragonesa [20], Lacaune [7] or Olkuska [17] mutations. However the effect reported here seems much higher than the effect of the Grivette mutation of the same *BMP15* gene [17]. The four known mutations in the *GDF9* gene in Belclare [19], Thoka [25], Santa Ines [6] and Norwegian White Sheep populations [26] also induce a slightly higher increase in LS in the heterozygous state than the *FecL* mutation. As for most mutations that do not induce sterility at the homozygous level, the prolificacy of homozygous LL ewes is too high and not useful for breeders.

The significant interaction between the set of thresholds and genotype can be interpreted as a double effect of the genotype, impacting the mean of LS and also its variability in addition to the systematic increase induced by the mean change. If the difference between prolificacy of ++ and L + ewes had resulted only in a simple shift in the mean of the underlying variable, as was observed between breeds by Bodin and Elsen [16], it would have induced a much lower increase in the observed variability of LS (see Additional file 1: Table S1); in particular, the percentage of twin lambing would have increased up to 64% in L + ewes compared to 51% actually observed (see Additional file 2: Figure S1). To our knowledge, this is the first time that a major gene controlling the mean of LS has been reported to have an additional effect on the variability of the trait. Due to the low number of LL ewes, we could not determine whether the same effect would be conserved if a second copy of the mutant allele was added.

This effect on the mean and the variability is perhaps not common to all major genes that affect LS in sheep and could result from specific physiological mechanisms that are regulated by the Lacaune gene. It is known that, in contrast to other mutations, the *FecL* mutation induces shorter delays of pre-ovulatory luteinizing hormone (LH) and follicle-stimulating hormone (FSH) surges after sponge withdrawal [27] and that treatments used for oestrus synchronisation (which also modify these events) have an important effect on the variability of LS [16]. Therefore, it would interesting to determine whether the LS variability is further increased in L + ewes that are synchronized with the classical synchronisation treatment (vaginal sponge impregnated with injections of fluorogestone acetate (FGA) and pregnant mare serum gonadotrophin (PMSG) at withdrawal) compared with non-treated L + ewes.

The estimates of the genetic parameters for prolificacy were in agreement with estimates reported in the literature from threshold models [15,28] or from linear models [29] and transformation using the formula given by Gianola [13]. However, in our case, the estimate of additive genetic variance, which was found to be towards the upper limit of the range of values found in the literature, included the permanent environmental variance, which could not be efficiently estimated in an independent manner. Although the estimate of heritability of LS obtained when ignoring the existence of the major gene was biased, the estimate was lower than the very high estimates found in the same Lacaune population in 1988 after 12 years of intensive selection for prolificacy (heritability = 0.37 [30]), or in 2001 after 25 years of selection (heritability = 0.39 [31]). The lower estimate obtained in this study results from the strict management rules that have been implemented over the last 12 years to prevent the gene from being disseminated too rapidly and, most of all, to avoid producing homozygous animals. The lower heritability also results from the elimination of the *BMP15 C53Y* mutation, which was still segregating in 2001 [7]. Finally, based on the estimate of the gene effect and its frequency in the current Lacaune population, the *FecL* gene accounts for a very large part (42%) of the total additive genetic variance and this could explain the rapid increase in genetic progress for LS observed in this population.

### Gene effect on fertility

The rate of AI success was very high in this data and higher than the value reported in dairy Lacaune sheep [32], although the latter strain is renowned for its very high AI results. In our data, the age of the ewes and the insemination periods were favourable and these factors, together with the fact that 2012 was a good year for AI fertility in Lacaune sheep, could explain this high success rate.

To our knowledge, this is first time that a major gene controlling prolificacy is reported to have an effect on fertility, except for some *BMP15* and *GDF9* mutations in the homozygous state [7,17-20,25]. This effect might be explained by the particular endocrine profile of LL ewes when compared with ewes that carry mutations in other major genes (*BMPR1B*, *BMP15*, *GDF9*) [27]. Indeed, after sponge withdrawal following a classical progestative treatment, FSH and LH surges appear 20 h earlier in LL ewes than in ++ ewes [27]. Although these results were observed without the PMSG injection, which normally follows the progestative treatment at the time of insemination, the difference in delay in the LH surge might explain the effect on fertility due to a difference in the optimum time for AI after sponge withdrawal. However, the higher fertility of L + ewes would indicate that the delay between the end of the treatment and AI is not optimal for ++ Lacaune ewes. Before changing this delay, it is necessary to accumulate and analyze more data that take other potential variation factors, which were not available in our study, into account [32].

### Liveweight at 30 days of age

The effect of the *FecL* genotype of the dams on the weight of their lambs at 30 days was quite surprising and no similar effects have ever been described for other major prolificacy genes. The data available did not enable us to determine whether the difference between lambs was due to weight at birth, growth between 0 and 30 days, or both. A difference in weight at birth could be due to a higher rate of embryonic losses in the carrier ewes. It has been observed that, in the event of embryonic losses, the remaining foetuses weigh less at birth than foetuses from litters of similar size but without embryonic losses [33]. Therefore, the possible influence of the dam’s genotype on embryonic mortality requires further study. Another hypothesis is that the difference in lamb weight at 30 days of age is due to different maternal abilities of the two genotypes. It can be assumed that such differences in dairy capacities exist but that they are not directly due to the effect of the *FecL* gene. In the breeding program used in this study, animals were selected on a genetic index, combining EBV for prolificacy and maternal ability. Thus, animals with very good prolificacy EBV were selected even if their dairy EBV was moderate, and conversely animals with a very high maternal ability value were selected even if their prolificacy value was slightly lower than average. Because the average prolificacy EBV of L + animals was higher than that of ++ ewes, it is possible that these L + ewes were selected despite their low maternal ability.

### Lamb mortality

The likelihood of death was higher for twin lambs born from L + dams than from ++ dams. This result was surprising. Indeed, the effect of birth type on lamb mortality is well documented [15,34], and the effect of the *FecL* gene most probably results from the higher ovulation rate and pregnancy wastage of L + ewes, as is the case for F + Booroola ewes [33]. However, there is no clear explanation for the interaction between dam genotype and litter size, except the low statistical power of the test due to the low number of triplets born from ++ ewes. If differences in mortality exist between the progenies of ++ and L + ewes, they are probably lower than those observed for litters of twins. In particular, for litter sizes of 1, for which a large amount of data is available for both genotypes, the gene effect is very low and the associated P value is high (P > 075). If this effect is confirmed, it would be necessary to genotype the dam and its lambs (even those that die before 30 days) in order to separate the maternal and direct effects of the gene.

### Prolificacy EBV

When the evaluation model included the effect of genotype, the EBV accounted only for the polygenic effects and, as expected, there was no difference between the ++ and the L + animals. In contrast, when the genotype was not included in the model, its effect was not dissociated from the polygenic effects and was included in the EBV, which led to much higher EBV for L + than for ++ ewes. For L + ewes, this bias in the estimates of the additive polygenic values, assessed by the difference between EBV and EBV|_FecL_, was very large and can contribute to biased selection for polygenic values. Furthermore, the increase of bias when the additive polygenic value of L + ewes increased, might express an interaction of the *FecL* mutation with the rest of the genome or more simply with another gene. Although carrier sires of the *BMP15* mutation are systematically discarded from breeding, this mutation is still present in the female population and might contribute to this interaction. The bias observed for ++ ewes was much lower, perhaps due to the high percentage of this genotype in the sample. For several reasons (economic superiority of heterozygous ewes, optimization of future genetic progress, etc.), the global genetic values should not be used for selection and the gene effect should be separated from the polygenic effects in the genetic evaluation. Before changing the entire evaluation system, a first improvement would be to estimate a pseudo polygenic EBV for animals with a known genotype. This pseudo polygenic EBV would be assessed by removing or adding the value of the genotype from the current individual EBV. However, in the future, the evaluation model will take the genotype effect into account, which will be estimated as in the present study. Individuals without known genotypes would be assigned a unique common genotype or individual probabilities of each genotype. Genotype probabilities can be calculated exactly using iterative peeling [35] when there is no inbreeding, or estimated in the case of a complex pedigree with many loops [36].

## Conclusions

The large amount of genotyping data accumulated for *FecL* in the Lacaune sheep of the Ovi-Test population since 2011 has enabled us to better estimate genotype frequencies and the effect of this gene on prolificacy and fertility (traits related to quantity and quality of ovulation), as well as other important traits for breeders. The negative effects of the maternal genotype on growth and mortality of lambs are nevertheless relatively low in comparison to the effects on litter size and fertility after AI. The increase in variability of litter size due to the *FecL* mutation is not a negative outcome, to the extent that triplets are accepted by breeders. However, since some breeders wish to limit the number of triplets and to avoid litter sizes greater than 3 at all cost, management of the *FecL* gene should be associated with a reduction of litter size variability, perhaps by canalyzing selection [37]. This paper also shows that ignoring the existence of a major gene in genetic evaluation could lead to a large overestimation of polygenic breeding values for prolificacy.

## Appendix

A threshold model of categorical traits supposes an underlying variable with a standard normal distribution and a set of thresholds which transforms this continuous variable into a multinomial variable with *j* ordered categories. However, any threshold model with two thresholds *Y* ~ N(0, 1) ; {*τ*_1_ , *τ*_2_} can be re-parameterized by a homothetic transformation that scales and shifts the set of thresholds to the values of a reference model and changes accordingly the parameters of the underlying variable.

Thus, two models:*Y*^*a*^ ~ N$\left(0,1\right)\;;\;\left\{\tau_{1}^{a}\;,\;\tau_{2}^{a}\right\}$ and *Y*^*b*^ ~ N$\left(0,1\right)\;;\;\left\{\tau_{1}^{b}\;,\;\tau_{2}^{b}\right\}$ are strictly equivalent to the two models: *Y*^*a*^ ~ N$\left(0,1\right)\;;\;\left\{\tau_{1}^{a}\;,\;\tau_{2}^{a}\right\}$ and *Y*^b*^ ~ N $\left(\eta ,\sigma \right)\;;\;\left\{\tau_{1}^{a}\;,\;\tau_{2}^{a}\right\}$, where $\sigma =\left(\tau_{2}^{a}-\tau_{1}^{a}\right)/\left(\tau_{2}^{b}-\tau_{1}^{b}\right)$ and $\eta =\tau_{1}^{a}-\sigma \;\tau_{1}^{b}$.

It is then possible to interpret the differences of these models having the same set of thresholds, by a shift *η* and a scaling effect *σ* of the underlying variable of the model for *Y*^b*^.

This homothetic transformation can be also used for re-parameterization of models with three thresholds if

$$\left(\tau_{2}^{a}-\tau_{1}^{a}\right)/\left(\tau_{2}^{b}-\tau_{1}^{b}\right)=\sigma =\left(\tau_{3}^{a}-\tau_{2}^{a}\right)/\left(\tau_{3}^{b}-\tau_{2}^{b}\right).$$

In other cases, an approximate re-parameterization can be done if

$$\left(\tau_{2}^{a}-\tau_{1}^{a}\right)/\left(\tau_{2}^{b}-\tau_{1}^{b}\right)\approx \left(\tau_{3}^{a}-\tau_{2}^{a}\right)/\left(\tau_{3}^{b}-\tau_{2}^{b}\right).$$

In this case, the values of the two first thresholds of the common new set can be those of the reference threshold set $\left\{\tau_{1}^{a}\;,\;\tau_{2}^{a}\;,\;\tau_{3}^{a}\right\}$, but the third is given by $\tau_{3}^{a*}=\sigma \left(\tau_{3}^{b}-\tau_{1}^{b}\right)-\tau_{1}^{a}$ with $\sigma =\left(\tau_{2}^{a}-\tau_{1}^{a}\right)/\left(\tau_{2}^{b}-\tau_{1}^{b}\right)$.

In our particular case, if one argues that the third threshold for the ++ genotype (corresponding to litters of more than three lambs) was poorly estimated (322 litters of three lambs in 4071 lambing; 7.91% ± 1.09), it was possible to change the parameters by an approximate re-parameterization (see Additional file 1: Table S1). This parameter transformation induced a small change of the third threshold for the ++ genotype that resulted in a negligible change of the estimated percent of triple lambing (from 5.36 to 5.61%).

By this transformation, we can interpret the effect of the gene as an increase of the mean and an 81% increase of the underlying variance for the L + genotype (see Additional file 1: Table S1). Figure S1 (see Additional file 2: Figure S1) shows the distribution of LS for ++ and L + genotypes with this approximate re-parameterization. Figure S1 (see Additional file 2: Figure S1) also shows the theoretical LS distribution of a population with an increased L + mean but a conserved ++ variance.

## Competing interests

The authors declare that they have no competing interests.

## Authors’ contributions

PM, JR and LB jointly designed the study and discussed the results. PM extracted, computed the data and performed the statistical analysis. PM wrote the first draft of the manuscript, which was then modified by LB and discussed with other co-authors. All authors read and approved the final manuscript.

## Supplementary Material

Additional file 1: Table S1 Estimated thresholds for the threshold animal mixed model and transformed thresholds†; resulting distribution, mean and variance of the litter size (LS). This table shows the estimated thresholds and the mean and variance of the underlying variable obtained with the threshold model and the resulting mean and variance of the litter size. It also shows the common set of transformed thresholds with the mean and the variance of the underlying variable, which results in the same distribution for litter size on the observable scale than the estimated thresholds. Click here for file

Additional file 2: Figure S1 Distribution of underlying normal variables and the transformed common set {τ_c_} of thresholds. The figure represents the common set of transformed thresholds and the underlying variables y^++^ ~ N(0,1) and y^L+^ ~ N$\left(\eta^{L+},\sigma^{2_{L+}}\right)$ for ++ and L + ewes, respectively. These distributions make it possible to predict the proportions of litter size (LS) for both genotypes. The figure also displays a theoretical underlying variable y^L+*^ ~ N(*η*^*L* +^, 1), with the same mean as for L + ewes and the same standardized residual variance as for ++ ewes and the resulting theoretical proportions of LS. Click here for file

## References

[B1] King JWB Fahmy MH Historical background and recognition of prolific sheep Prolific sheep 1996 Wallingford: Cab International 3 7

[B2] Bodin L SanCristobal M Lecerf F Mulsant P Bibe B Lajous D Belloc JP Eychenne F Amigues Y Elsen JM Segregation of a major gene influencing ovulation in progeny of Lacaune meat sheep Genet Sel Evol 2002 34 447 464 12270104 10.1186/1297-9686-34-4-447 PMC2705455

[B3] Piper LR Bindon BM Genetic segregation for fecundity in Booroola Merino sheep Proceedings of the First World Congress on Sheep and Beef Cattle Breeding: 28 October - 13 November 1980; Palmerston North 1982 395 400

[B4] Davis GH Montgomery GW Allison AJ Kelly RW Bray AR Segregation of a major gene Influencing fecundity in progeny of Booroola sheep New Zeal J Agr Res 1982 25 525 529

[B5] Bodin L Raoul J Demars J Drouilhet L Mulsant P Sarry J Tabet C Tosser-Klopp G Fabre S Boscher MY Tiphine L Bertrand C Bouquet PM Maton C Teyssier J Jouannaux C Hallauer J Cathalan D Gueux J Pocachard M Etat des lieux et gestion pratique des gènes d'ovulation détectés dans les races ovines françaises18èmes Rencontres Recherches Ruminants: 7–8 Décembre 2011; Paris 2011 393 400

[B6] Silva BDM Castro EA Souza CJH Paiva SR Sartori R Franco MM Azevedo HC Silva T Vieira AMC Neves JP Melo EO A new polymorphism in the Growth and Differentiation Factor 9 (GDF9) gene is associated with increased ovulation rate and prolificacy in homozygous sheep Anim Genet 2011 42 89 92 20528846 10.1111/j.1365-2052.2010.02078.x

[B7] Bodin L Di Pasquale E Fabre S Bontoux M Monget P Persani L Mulsant P A novel mutation in the bone morphogenetic protein 15 gene causing defective protein secretion is associated with both increased ovulation rate and sterility in Lacaune sheep Endocrinology 2007 148 393 400 17038554 10.1210/en.2006-0764

[B8] Drouilhet L Lecerf F Bodin L Fabre S Mulsant P Fine mapping of the FecL locus influencing prolificacy in Lacaune sheep Anim Genet 2009 40 804 812 19466934 10.1111/j.1365-2052.2009.01919.x

[B9] Drouilhet L Mansanet C Sarry J Tabet K Bardou P Woloszyn F Lluch J Harichaux G Viguie C Monniaux D Bodin L Mulsant P Fabre S The highly prolific phenotype of Lacaune sheep is associated with an ectopic expression of the B4GALNT2 gene within the ovary PLoS Genet 2013 9 e1003809 24086150 10.1371/journal.pgen.1003809 PMC3784507

[B10] Bodin L Lecerf F Bessière M Mulsant P Features of major genes for ovulation in the *Lacaune* population Proceedings of the 8th World Congress on Genetics Applied to Livestock Production; 13–18 August 2006; Belo Horizonte 2006 communication 15–05

[B11] Fisher RA The correlation between relatives on the supposition of mendelian inheritance Philos T Roy Soc Edinburgh 1918 52 399 433

[B12] Gilmour AR Gogel BJ Cullis BR Thompson R ASReml User Guide Release 3.0. VSN 2009 VSN International Ltd: Hemel Hempstead

[B13] Gianola D Theory and analysis of threshold characters J Anim Sci 1982 54 1079 1096

[B14] Misztal I Gianola D Foulley JL Computing aspects of a nonlinear method of sire evaluation for categorical data J Dairy Sci 1989 72 1557 1568

[B15] Matos CAP Thomas DL Gianola D Tempelman RJ Young LD Genetic analysis of discrete reproductive traits in sheep using linear and nonlinear models.1. Estimation of genetic parameters J Anim Sci 1997 75 76 87 9027551 10.2527/1997.75176x

[B16] Bodin L Elsen JM Variability of litter size of French sheep breeds following natural or induced ovulation Anim Prod 1989 48 535 541

[B17] Demars J Fabre S Sarry J Rossetti R Gilbert H Persani L Tosser-Klopp G Mulsant P Nowak Z Drobik W Martyniuk E Bodin L Genome-wide association studies identify two novel BMP15 mutations responsible for an atypical hyperprolificacy phenotype in sheep PLoS Genet 2013 9 e1003482 23637641 10.1371/journal.pgen.1003482 PMC3636084

[B18] Galloway SM McNatty KP Cambridge LM Laitinen MPE Juengel JL Jokiranta TS McLaren RJ Luiro K Dodds KG Montgomery GW Beattie AE Davis GH Ritvos O Mutations in an oocyte-derived growth factor gene (BMP15) cause increased ovulation rate and infertility in a dosage-sensitive manner Nat Genet 2000 25 279 283 10888873 10.1038/77033

[B19] Hanrahan JP Gregan SM Mulsant P Mullen M Davis GH Powell R Galloway SM Mutations in the genes for oocyte-derived growth factors GDF9 and BMP15 are associated with both increased ovulation rate and sterility in Cambridge and Belclare sheep (Ovis aries) Biol Reprod 2004 70 900 909 14627550 10.1095/biolreprod.103.023093

[B20] Monteagudo LV Ponz R Tejedor MT Lavina A Sierra I A 17 bp deletion in the Bone Morphogenetic Protein 15 (BMP15) gene is associated to increased prolificacy in the Rasa Aragonesa sheep breed Anim Reprod Sci 2009 110 139 146 18282670 10.1016/j.anireprosci.2008.01.005

[B21] Bindon BM Piper LR Cummins LJ O'Shea T Hillard MA Findlay JK Robertson DM Land RB, Robinson DW Reproductive endocrinology of prolific sheep: studies of the Booroola Merino Genetics of Reproduction in Sheep 1985 London: Butterworths 217 235

[B22] Fogarty NM Environmental modulation of *FecB* expression International Workshop on Booroola Gene; 10–12 November 2008; Pune 2008

[B23] Davis GH McEwan JC Fennessy PF Dodds KG Farquhar PA Evidence for the presence of a major gene influencing ovulation rate on the X chromosome of sheep Biol Reprod 1991 44 620 624 2043732 10.1095/biolreprod44.4.620

[B24] Davis GH McEwan JC Fennessy PF Dodds KG McNatty KP O WS Infertility due to bilateral ovarian hypoplasia in sheep homozygous (FecXIFecXI) for the Inverdale prolificacy gene located on the X chromosome Biol Reprod 1992 46 636 640 1533540 10.1095/biolreprod46.4.636

[B25] Nicol L Bishop SC Pong-Wong R Bendixen C Holm LE Rhind SM McNeilly AS Homozygosity for a single base-pair mutation in the oocyte-specific GDF9 gene results in sterility in Thoka sheep Reproduction 2009 138 921 933 19713444 10.1530/REP-09-0193

[B26] Vage DI Husdal M Kent MP Klemetsdal G Boman IA A missense mutation in growth differentiation factor 9 (GDF9) is strongly associated with litter size in sheep BMC Genet 2013 14 1 23280002 10.1186/1471-2156-14-1 PMC3546915

[B27] Drouilhet L Taragnat C Fontaine J Duittoz A Mulsant P Bodin L Fabre S Endocrine characterization of the reproductive axis in highly prolific Lacaune sheep homozygous for the FecL(L) mutation Biol Reprod 2010 82 815 824 20075395 10.1095/biolreprod.109.082065

[B28] Janssens S Vandepitte W Bodin L Genetic parameters for litter size in sheep: natural versus hormone-induced oestrus Genet Sel Evol 2004 36 543 562 15339632 10.1186/1297-9686-36-5-543 PMC2697192

[B29] Baelden M Tiphine L Poivey JP Bouix J Bibe B Robert-Granie C Bodin L Estimation of genetic parameters for litter size after natural and hormone-induced oestrus in sheep Livest Prod Sci 2005 97 275 281

[B30] Bodin L Bibe B Blanc MR Ricordeau G Genetic relationship between prepuberal plasma FSH levels and reproductive performance in Lacaune ewe lambs Genet Sel Evol 1988 20 489 498 22879342 10.1186/1297-9686-20-4-489 PMC2712511

[B31] SanCristobal-Gaudy M Bodin L Elsen JM Chevalet C Genetic components of litter size variability in sheep Genet Sel Evol 2001 33 249 271 11403747 10.1186/1297-9686-33-3-249 PMC2705407

[B32] David I Robert-Granie C Manfredi E Lagriffoul G Bodin L Environmental and genetic variation factors of artificial insemination success in French dairy sheep Animal 2008 2 979 986 22443696 10.1017/S1751731108002152

[B33] Hinch GN Kelly RW Davis GH Owens JL Crosbie SF Factors affecting lamb birth weights from high fecundity Booroola ewes Anim Reprod Sci 1985 8 53 60

[B34] Gama LT Dickerson GE Young LD Leymaster KA Genetic and phenotypic variation in sources of preweaning lamb mortality J Anim Sci 1991 69 2744 2753 1885386 10.2527/1991.6972744x

[B35] Fernando RL Stricker C Elston RC An efficient algorithm to compute the posterior genotypic distribution for every member of a pedigree without loops Theor Appl Genet 1993 87 89 93 24190198 10.1007/BF00223750

[B36] Vitezica ZG Legarra A Accuracy of genotype estimation using loop breakers Proceedings of the 8th World Congress on Genetics Applied to Livestock Production; 13–18 August 2006; Belo Horizonte 2006 communication 20–13

[B37] Scheiner SM Lyman RF The genetics of phenotypic plasticity.1. Heritability J Evol Biol 1989 2 95 107

