# Supplementary material for: Effects of the FecL major gene in the Lacaune meat sheep population
Source: Genet Sel Evol. 2014 Aug 12;46(1):48. doi: 10.1186/1297-9686-46-48 (PMC4237826; doi:10.1186/1297-9686-46-48)
Supplement: Additional file 1: Table S1 — Estimated thresholds for the threshold animal mixed model and transformed thresholds†; resulting distribution, mean and variance of the litter size (LS). This table shows the estimated thresholds and the mean and variance of the underlying variable obtained with the threshold model and the resulting mean and variance of the litter size. It also shows the common set of transformed thresholds with the mean and the variance of the underlying variable, which results in the same distribution for litter size on the observable scale than the estimated thresholds. [file 1297-9686-46-48-S1.docx]

**Additional file 1: Table S1**

**Table S1: Estimated thresholds of the threshold animal mixed model and transformed thresholds^†^; resulting distribution, mean and variance of LS**

|  | **Estimated thresholds** | | **Transformed thresholds** | |
| --- | --- | --- | --- | --- |
| Thresholds | **++** | **L+** | **++** | **L+** |
| τ_1_ | -0.287 | -0.849 | -0.287 | |
| τ_2_ | 1.581 | 0.539 | 1.581 | |
| τ_3_ | 2.711 | 1.691 | 3.131 | |
| Underlying variable parameters |  |  |  | |
| η | 0 | 0 | 0 | 0.86 |
| σ² | 1 | 1 | 1 | 1.81 |
| Resulting parameters on the observable scale |  |  |  |  |
| μ_obs_ | 1.67 | 2.14 | 1.67 | 2.14 |
| σ²_obs_ | 0.35 | 0.61 | 0.34 | 0.61 |
| %LS1 | 38.71 | 19.79 | 38.71 | 12.55 |
| %LS2 | 55.60 | 50.71 | 55.60 | 63.88 |
| %LS3 | 5.36 | 24.95 | 5.61 | 22.42 |
| %LS4+ | 0.34 | 4.54 | 0.09 | 1.16 |

Values in columns "estimated thresholds" correspond to the output of the threshold model: thresholds (τ1− τ3), mean and variance of the underlying variable (η, σ²) and the resulting mean and variance (µ_obs,_ σ²_obs_) of the litter size % (%LS1 - %LS4+) on the observable scale. Values for "transformed thresholds" columns correspond to a common set of thresholds for both genotypes and a change of the mean and the variance of the underlying variable for L+ which provides the same distribution of LS on the observable scale than the estimated thresholds.
