# Supplementary material for: Effects of the FecL major gene in the Lacaune meat sheep population
Source: Genet Sel Evol. 2014 Aug 12;46(1):48. doi: 10.1186/1297-9686-46-48 (PMC4237826; doi:10.1186/1297-9686-46-48)
Supplement: Additional file 2: Figure S1 — Distribution of underlying normal variables and the transformed common set {τc} of thresholds. The figure represents the common set of transformed thresholds and the underlying variables y++ ~ N(0,1) and yL+ ~ NηL+,σ2L+ for ++ and L + ewes, respectively. These distributions make it possible to predict the proportions of litter size (LS) for both genotypes. The figure also displays a theoretical underlying variable yL+* ~ N(ηL +, 1), with the same mean as for L + ewes and the same standardized residual variance as for ++ ewes and the resulting theoretical proportions of LS. [file 1297-9686-46-48-S2.docx]

**Additional file 2 Figure S1**

**
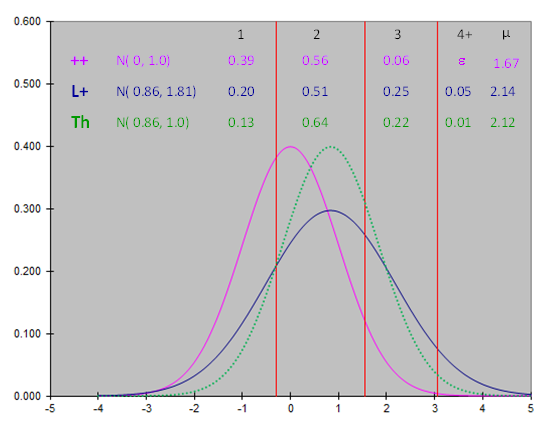
Figure S1: Distribution of underlying normal variables and the transformed common set {τ^c^} of thresholds**

The purple line represents the underlying variable y^++^ ~ N(0,1) providing the estimated proportions of LS in ++ ewes; the blue line represents the transformed underlying variable y^L+^ ~ N$(\eta^{L+},\sigma^{2_{L+}})$ for L+ ewes providing the estimated proportions of LS in L+ ewes; the green-dotted line represents a theoretical underlying variable y^L+*^ ~ N$(\eta^{L+},1)$, with the same mean as for L+ ewes and a standardized residual variance as for ++ ewes.
